# Can changes in health related quality of life scores predict survival in stages III and IV colorectal cancer?

**DOI:** 10.1186/1477-7525-9-62

**Published:** 2011-08-03

**Authors:** Donald P Braun, Digant Gupta, James F Grutsch, Edgar D Staren

**Affiliations:** 1 Office of Clinical Research, Cancer Treatment Centers of America® (CTCA) at Midwestern Regional Medical Center, 2520 Elisha Ave., Zion, IL, 60099, USA

## Abstract

**Background:**

Several studies have demonstrated the predictive significance on survival of baseline quality of life (QoL) in colorectal cancer (CRC) with little information on the impact of changes in QoL scores on prognosis in CRC. We investigated whether changes in QoL during treatment could predict survival in CRC.

**Methods:**

We evaluated 396 stages III-IV CRC patients available for a minimum follow-up of 3 months. QoL was evaluated at baseline and after 3 months of treatment using EORTC QLQ-C30. Cox regression evaluated the prognostic significance of baseline, 3-month and changes in QoL scores after adjusting for age, gender and stage at diagnosis.

**Results:**

After adjusting for covariates, every 10-point increase in both baseline appetite loss and global QoL score was associated with a 7% increased risk of death with HR = 1.07 (95% CI, 1.01-1.14; *P *= 0.02) and (HR = 0.93 (95% CI, 0.87-0.98; *P *= 0.01) respectively. A lower risk of death was associated with a 10-point improvement in physical function at 3 months (HR, 0.86; 95% CI, 0.78-0.94; *P *= 0.001). Surprisingly, a higher risk of death was associated with a 10-point improvement in social function at 3 months (HR, 1.08; 95% CI, 1.02-1.13; *P *= 0.008).

**Conclusions:**

This study provides preliminary evidence to indicate that CRC patients whose physical function improves within 3 months of treatment have a significantly increased probability of survival. These findings should be used in clinical practice to systematically address QoL-related problems of CRC patients throughout their treatment course.

## Background

Quality of life (QoL) is a multidimensional construct. A growing consensus among health care providers and researchers is that treatment efficacy should be judged by effects on both quantity and quality of life; this has led to the inclusion of QoL assessment as a primary endpoint in cancer clinical trials along with traditional endpoints of tumor response and survival. There is general agreement in the medical and scientific research community that patients are the best source of information regarding their QoL. Consequently, the use of self-reported QoL assessment has become a valuable tool for both clinical practice and research. There are extensive data in the literature demonstrating that pretreatment/baseline QoL can predict survival in several different types of cancers independent of the extent of the disease and other clinical prognostic factors [1-10], however, evidence is only beginning to emerge regarding the prognostic significance of changes in QoL scores in cancer [11-15].

Advanced stage colorectal cancer (CRC) is associated with significant morbidity, which when coupled with the adverse effects of cancer treatment, can further deteriorate patient QoL. A few studies have evaluated the relationship between pretreatment QoL and survival in CRC [7,16-19]. However, to the best of our knowledge, there is no study in the literature investigating the prognostic significance of changes in QoL scores in CRC. In the current study, we investigated whether pretreatment QoL parameters as well as changes in QoL scores from baseline until 3 months after treatment could predict survival in patients with stages III-IV CRC.

## Methods

### Study Population

We examined 396 histologically confirmed stages III and IV colorectal cancer patients treated at Cancer Treatment Centers of America^® ^at Midwestern (MRMC) and Southwestern (SRMC) Regional Medical Centers between January 2001 and December 2009. None of these patients had received any treatment at our hospitals when contacted to participate in this investigation. The inclusion criteria for participation in this study were a histological diagnosis of stage III or IV colorectal cancer and the ability to read English. Patients were excluded if they were unable to give informed consent or were unable to understand or cooperate with study conditions.

A trained clinical coordinator was responsible for determining eligibility, describing the study, and obtaining informed consent. All patients were assured that refusal to participate would not affect their future care in any way. Patients who chose to participate were presented with the QoL questionnaire at their initial/baseline visit and instructed to return their completed questionnaires to the clinical coordinator within 24 hours. Thus, patients completed baseline QoL questionnaires prior to receiving therapy at our facility. Following the completion of the baseline questionnaire, all patients were treated with an integrative model combining surgery, radiation and chemotherapy as appropriate, plus complementary therapy consisting primarily of nutritional, psychosocial, and spiritual support, naturopathic supplements, pain management, and physical therapy/rehabilitation.

Additional data recorded for this study included age at diagnosis, gender, stage of disease at diagnosis (III versus IV) and prior treatment history (previously treated versus newly diagnosed). The only follow-up information required was the date of death or the date of last contact/last known to be alive, obtained from the tumor registries at MRMC and SRMC. This study was approved by the Institutional Review Board at Cancer Treatment Centers of America^®^.

### QoL Assessment

QoL was assessed at baseline and after 3 months of treatment using the European Organization for the Research and Treatment of Cancer Quality of Life Questionnaire (EORTC QLQ-C30), which emphasizes a patient's capacity to fulfill the activities of daily living. The EORTC QLQ-C30 is a 30-item cancer specific questionnaire that incorporates five functioning scales (physical, role, cognition, emotional, and social), eight symptom scales (fatigue, pain, and nausea/vomiting, dyspnea, insomnia, loss of appetite, constipation, diarrhea, financial problems), financial well-being scale and a global scale (based on two items: global health and global QoL). The raw scores are linearly transformed to give standard scores in the range of 0-100 for each of the functioning and symptom scales. Higher scores in the global and functioning scales and lower scores in the symptom scales indicate better QoL. A difference of 5-10 points in the scores represents a small change, 10-20 points a moderate change and greater than 20 points a large, clinically significant change from the patient's perspective [20]. This instrument has been extensively tested for reliability and validity [21-23].

### Statistical Analysis

Patient survival was the primary end point and defined as the time interval between the date of first patient visit to the hospital and the date of death from any cause or the date of last contact/last known to be alive. Two separate analyses were performed. First, the relationship between baseline QoL and patient survival was investigated for 396 patients. Second, the relationship between change in QoL scores between baseline and 3 months and survival was assessed for the same patient cohort. Change scores were calculated by subtracting baseline from 3-month QoL scores. The overall survival was calculated using the Kaplan-Meier method. Clinical and QoL variables were evaluated using univariate Cox proportional hazards models to determine which parameters showed individual prognostic value for survival. Multivariate Cox proportional hazards models were then performed to evaluate the joint prognostic significance of all QoL and clinical factors.

In order to minimize instability of the final multivariate model resulting from high multicollinearity, global QoL was evaluated separately because it is most highly correlated with all other variables on the EORTC QLQ-C30 questionnaire, and also because it is difficult to interpret and manipulate clinically [24]. Each EORTC QLQ-C30 scale was treated as a continuous variable for the purpose of Cox regression analyses. The effect of QoL parameters on patient survival was expressed as hazard ratios (HRs) with 95% confidence intervals (CIs). Changes of 10 or more points on a 0 to100 scale are considered clinically relevant [20], so we present HRs for a 10-point change on the continuous QoL variables. An effect was considered to be statistically significant if the p value was less than or equal to 0.05. All statistical tests were two sided. All data were analyzed using SPSS version 17.0 (SPSS, Chicago, IL, USA).

Cox regression with time-invariant covariates assumes that the ratio of hazards for any two groups remains constant in proportion over time. We checked this assumption by first examining log-minus-log plots for the categorical predictors and then fitting a Cox regression with a time-varying covariate for each predictor in turn. Potential multicollinearity was assessed using multiple approaches. Large values (above 0.75) of Pearson's correlation coefficients were used as an initial screen for pairs of QoL variables, with one member of the pair not entered into the multivariate model (the measure that was more meaningful or actionable was retained). As a second check, the variance inflation factor (VIF) was used with the final model to verify that multicollinearity was not significantly influencing model coefficients [25,26]. Finally, the possible influence of sample bias and multicollinearity on the results was investigated using a bootstrap re-sampling procedure. We generated 500 samples, each the same size as the original data set, by random selection with replacement. Cox regression was then run separately on these 500 samples to obtain robust estimates of the standard errors of coefficients, and hence the p values and confidence intervals of the model coefficients [27].

## Results

### Patient Characteristics

Table 1 describes the baseline characteristics of our patient cohort. At the time of this analysis, 211 deaths had occurred among the 396 participants. Table 2 describes the results of univariate Cox regression analysis for baseline patient characteristics. Stage at diagnosis and prior treatment history were significantly associated with survival while age at diagnosis and gender were not. Median overall survival for the entire patient cohort was 16.2 months (95% CI: 13.0-19.4 months). The median survival for newly diagnosed and previously treated disease was 32.3 and 12.9 months respectively, p < 0.001. The median survival for patients with stage III and stage IV disease was 16.9 and 15.8 months respectively, p = 0.009.

**Table 1:** Baseline characteristics of 396 colorectal cancer patients

Characteristic	Categories	Number	Percent
Age at Diagnosis (years)	■ Mean	53.2	
	■ Median	54	
	■ Range	23-83	

Gender	■ Male	213	53.8
	■ Female	183	46.2

Vital Status	■ Death	211	53.3
	■ Alive	185	46.7

Treatment History	■ Newly diagnosed	120	30.3
	■ Previously treated	276	69.7

Stage at Diagnosis	■ Stage III	176	44.4
	■ Stage IV	220	55.6

**Table 2:** Baseline Characteristics and Associated HRs for Death

Characteristic	HR (95% CI)	P
Age at Diagnosis (years) used as continuous variable*	1.08 (0.94 - 1.21)	0.25

Gender (male as reference)	0.83 (0.63 - 1.1)	0.17

Treatment History (newly diagnosed as reference)	2.6 (1.9 - 3.6)	< 0.001*

Stage at Diagnosis (stage III as reference)	1.4 (1.1 - 1.9)	0.009*

HRs correspond to a 10-point increment for age; *P < 0.05

#### Association between Baseline QoL and Survival

Table 3 describes the baseline scores for all dimensions of EORTC QLQ-C30 instrument. Among the EORTC QLQ-C30 functioning scales, social functioning had the lowest (worst) mean score of 68.4 while the highest (best) mean score of 79.7 was recorded for cognitive functioning. Among the EORTC QLQ-C30 symptom scales, nausea/vomiting had the lowest (best) mean score of 13.4 while the highest (worst) mean score of 38.8 was recorded for fatigue. Table 3 also displays the results of univariate and multivariate Cox regression analyses for each QoL variable. The HRs along with their 95% CIs for every 10-point increase in all EORTC QLQ-C30 scales are given. On univariate analysis, baseline QoL variables that were predictive of survival were social function, dyspnea, loss of appetite, diarrhea and global health. Before proceeding with multivariate analysis, we checked the bivariate Pearson's correlation among the QoL variables to screen for observable multicollinearity. Role function and fatigue were highly correlated (Pearson's r = -0.80). It was decided to retain fatigue and discard role function in the multivariate model. This is because questions used in the fatigue scale are more directly related to a patient's illness and physical condition than those used in the role function scale. On multivariate analysis, only appetite loss was found to be significantly associated with survival such that every 10-point increase in baseline appetite loss score was associated with a 7% increased risk of death (HR, 1.07; 95% CI, 1.01 to 1.14; *P *= 0.02). In addition, age, gender, stage at diagnosis and prior treatment history were all found to be statistically significant in the multivariate model. A separate multivariate model was run for global QoL after adjusting for age, gender, stage and prior treatment history. It was found that every 10-point increase in baseline global QoL score was associated with a 7% decreased risk of death (HR, 0.93; 95% CI, 0.87 to 0.98; *P *= 0.01). VIF values for baseline QoL variables ranged from 1.1 (diarrhea) to 4.0 (fatigue), none of which indicates a significant problem with multicollinearity [25,26]. There was no evidence of non-proportional hazards in the multivariate models presented.

**Table 3:** Baseline QoL Measures and Associated HRs for Death

Baseline Variable	QoL Score Mean (SD)	Univariate		Multivariate	
		
		HR (95% CI)	P	HR (95% CI)	P
**General Quality of Life**					

Global	62.6 (24.0)	0.92 (0.87 - 0.98)	0.008*	0.93 (0.87 - 0.98)	0.01*

**General Function**					

Physical	78.6 (20.7)	0.96 (0.89 - 1.02)	0.16	1.08 (0.97 - 1.20)	0.14

Role	70.3 (30.3)	0.97 (0.92 - 1.02)	0.21	Not used	

Emotional	70.6 (22.7)	1.0 (0.94 - 1.06)	0.92	1.01 (0.92 - 1.09)	0.86

Cognitive	79.7 (22.0)	0.99 (0.94 - 1.05)	0.85	1.02 (0.93 - 1.12)	0.61

Social	68.4 (31.1)	0.96 (0.91 - 1.0)	0.04*	0.93 (0.86 - 1.01)	0.08

**General Symptom**					

Fatigue	38.8 (27.9)	1.04 (0.99 - 1.08)	0.14	1.01 (0.90 - 1.11)	0.91

Nausea/Vomiting	13.4 (22.3)	0.99 (0.93 - 1.05)	0.84	0.95 (0.87 - 1.03)	0.18

Pain	29.3 (30.6)	1.03 (0.99 - 1.08)	0.12	1.02 (0.95 - 1.08)	0.64

Dyspnea	19.5 (26.2)	1.06 (1.01 - 1.11)	0.02*	1.05 (0.99 - 1.12)	0.09

Insomnia	33.7 (31.8)	1.03 (0.99 - 1.07)	0.17	1.04 (0.99 - 1.10)	0.12

Appetite Loss	25.2 (31.2)	1.05 (1.0 - 1.09)	0.03*	1.07 (1.01 - 1.14)	0.02*

Constipation	17.5 (27.4)	0.96 (0.91 - 1.02)	0.17	0.94 (0.88 - 1.00)	0.06

Diarrhea	15.4 (24.1)	1.07 (1.02 - 1.12)	0.01*	1.01 (0.95 - 1.07)	0.64

Financial	32.5 (32.9)	0.99 (0.95 - 1.03)	0.67	0.98 (0.92 - 1.03)	0.44

• HRs correspond to a 10-point increment for QoL scores

• 2 sets of multivariate models were constructed: one for global QoL and other for all general function and symptom variables combined

• Multivariate model (for general function and symptom variables combined) adjusted for age, gender, stage at diagnosis, prior treatment history and all baseline QoL variables excepting role function

• Multivariate model for global QoL adjusted for age, gender, stage at diagnosis and prior treatment history

• *P < 0.05

In order to further investigate the stability of the classical multivariate Cox models reported in Table 3, we conducted a bootstrap re-sampling procedure based on 500 samples. The bootstrap estimates of the multivariate HRs along with corresponding p values and confidence intervals are provided in Table 4. For the most part, the p values for the coefficients for classical Cox regression and bootstrap Cox regression led to the same conclusion, except for the appetite loss scale, which although significant in the classical model, became marginally significant in the bootstrap model.

**Table 4:** Bootstrap Multivariate HRs for Baseline QoL Measures

Baseline Variable	HR (95% CI)	P
**General Quality of Life**		

Global	0.93 (0.87 - 0.98)	0.01*

**General Function**		

Physical	1.08 (0.97 - 1.22)	0.16

Role	Not used	

Emotional	1.01 (0.92 - 1.10)	0.86

Cognitive	1.02 (0.93 - 1.13)	0.57

Social	0.93 (0.83 - 1.02)	0.14

**General Symptom**		

Fatigue	1.01 (0.90 - 1.10)	0.87

Nausea/Vomiting	0.95 (0.83 - 1.05)	0.30

Pain	1.02 (0.95 - 1.09)	0.69

Dyspnea	1.05 (0.99 - 1.13)	0.14

Insomnia	1.04 (0.99 - 1.12)	0.19

Appetite Loss	1.07 (1.0 - 1.16)	0.06

Constipation	0.94 (0.85 - 1.01)	0.08

Diarrhea	1.01 (0.94 - 1.09)	0.73

Financial	0.98 (0.91 - 1.05)	0.48

• HRs correspond to a 10-point increment for QoL scores

• 2 sets of multivariate models were constructed: one for global QoL and other for all general function and symptom variables combined

• Multivariate model (for general function and symptom variables combined) adjusted for age, gender, stage at diagnosis, prior treatment history and all baseline QoL variables excepting role function

• Multivariate model for global QoL adjusted for age, gender, stage at diagnosis and prior treatment history

• *P < 0.05

#### Association between Changes in QoL and Survival

Table 5 describes the change in scores from baseline to 3 months for all dimensions of EORTC QLQ-C30 instrument. On average, they were small. Table 4 also displays the results of univariate and multivariate Cox regression analyses for change in QoL scores. On univariate analysis, none of the change variables was significantly predictive of survival. Before proceeding with multivariate analysis, we checked the bivariate Pearson's correlation among the change scores to screen for observable multicollinearity. Once again, change in role function scores and change in fatigue scores were highly correlated (Pearson's r = -0.73). It was decided to retain change in fatigue scores and discard change in role function scores in the multivariate model for the same reasons mentioned above. On multivariate analysis, change variables that were significantly predictive of survival were physical function and social function. A lower risk of death was associated with a 10-point improvement in physical function at 3 months after treatment (HR, 0.86; 95% CI, 0.78 to 0.94; *P *= 0.001). Surprisingly, a higher risk of death was associated with a 10-point improvement in social function at 3 months after treatment (HR, 1.08; 95% CI, 1.02 to 1.13; *P *= 0.008). In addition, age, stage at diagnosis and prior treatment history were found to be statistically significant in the multivariate model, while gender was not. A separate multivariate model was run for change in global QoL after adjusting for age, gender, stage and prior treatment history, but change in global QoL was not a significant predictor of survival. VIF values for change in QoL variables ranged from 1.1 (change in diarrhea) to 3.3 (change in fatigue), none of which indicates a significant problem with multicollinearity. There was no evidence of non-proportional hazards in the multivariate models presented.

**Table 5:** Change in QoL Measures and Associated HRs for Death

Change Variable	QoL Change Mean (SD)	Univariate		Multivariate	
		
		HR (95% CI)	P	HR (95% CI)	P
**General Quality of Life**					

Global	-1.8 (29.0)	1.00 (0.95 - 1.04)	0.93	0.99 (0.95 - 1.04)	0.81

**General Function**					

Physical	-2.0 (24.5)	0.96 (0.91 - 1.01)	0.14	0.86 (0.78 - 0.94)	0.001*

Role	-3.1 (38.3)	1.02 (0.99 - 1.05)	0.25	Not used	

Emotional	1.5 (28.9)	1.00 (0.96 - 1.04)	0.99	1.01 (0.95 - 1.07)	0.70

Cognitive	-0.50 (27.9)	1.01 (0.96 - 1.05)	0.69	0.99 (0.92 - 1.06)	0.74

Social	0.84 (36.9)	1.03 (1.00 - 1.07)	0.08	1.08 (1.02 - 1.13)	0.008*

**General Symptom**					

Fatigue	1.7 (34.3)	0.99 (0.95 - 1.02)	0.50	0.98 (0.90 - 1.05)	0.55

Nausea/Vomiting	2.0 (29.4)	1.01 (0.97 - 1.06)	0.61	1.03 (0.97 - 1.09)	0.39

Pain	-1.6 (37.2)	1.00 (0.96 - 1.03)	0.95	1.01 (0.96 - 1.07)	0.68

Dyspnea	0.34 (32.4)	0.98 (0.94 - 1.02)	0.38	0.98 (0.93 - 1.03)	0.46

Insomnia	1.9 (40.8)	1.00 (0.97 - 1.04)	0.88	1.0 (0.95 - 1.04)	0.84

Appetite Loss	0.76 (37.9)	0.98 (0.95 - 1.02)	0.38	0.96 (0.91 - 1.01)	0.12

Constipation	0.25 (34.2)	1.02 (0.99 - 1.06)	0.19	1.02 (0.98 - 1.07)	0.29

Diarrhea	1.9 (33.7)	0.99 (0.94 - 1.03)	0.56	1.02 (0.98 - 1.07)	0.26

Financial	3.0 (39.9)	1.00 (0.97 - 1.04)	0.78	1.01 (0.97 - 1.04)	0.79

• HRs correspond to a 10-point increment for QoL scores

• 2 sets of multivariate models were constructed: one for global QoL and other for all general function and symptom variables combined

• Multivariate model (for change in general function and symptom variables combined) adjusted for age, gender, stage at diagnosis, prior treatment history and all QoL change variables excepting role function change

• Multivariate model for change in global QoL adjusted for age, gender, stage at diagnosis and prior treatment history

• *P < 0.05

In order to further investigate the stability of the classical multivariate Cox models reported in Table 5 as well as the unexpected direction of association between social function change and survival, we conducted a bootstrap re-sampling procedure based on 500 samples. The bootstrap estimates of the multivariate HRs along with corresponding p values and confidence intervals are provided in Table 6. We found no significant differences in the coefficients p values between classical Cox regression and bootstrap Cox regression models. Physical function and social function change variables which were significant in the classical Cox model retained their significance in the bootstrap Cox model as well.

**Table 6:** Bootstrap Multivariate HRs for Change in QoL Measures

Change Variable	HR (95% CI)	P
**General Quality of Life**		

Global	0.99 (0.94 - 1.05)	0.85

**General Function**		

Physical	0.86 (0.75 - 0.95)	0.004*

Role	Not used	

Emotional	1.01 (0.95 - 1.09)	0.71

Cognitive	0.99 (0.91 - 1.06)	0.75

Social	1.08 (1.02 - 1.14)	0.01*

**General Symptom**		

Fatigue	0.98 (0.89 - 1.06)	0.55

Nausea/Vomiting	1.03 (0.95 - 1.10)	0.44

Pain	1.01 (0.94 - 1.07)	0.74

Dyspnea	0.98 (0.91 - 1.05)	0.53

Insomnia	1.0 (0.95 - 1.05)	0.82

Appetite Loss	0.96 (0.90 - 1.02)	0.15

Constipation	1.02 (0.98 - 1.08)	0.36

Diarrhea	1.02 (0.98 - 1.09)	0.36

Financial	1.01 (0.96 - 1.05)	0.78

• HRs correspond to a 10-point increment for QoL scores

• 2 sets of multivariate models were constructed: one for global QoL and other for all general function and symptom variables combined

• Multivariate model (for change in general function and symptom variables combined) adjusted for age, gender, stage at diagnosis, prior treatment history and all QoL change variables excepting role function change

• Multivariate model for change in global QoL adjusted for age, gender, stage at diagnosis and prior treatment history

• *P < 0.05

## Discussion

The current study was undertaken to investigate whether baseline QoL as well as changes in QoL after 3 months of treatment could predict survival in stages III and IV CRC. We chose EORTC QLQ-C30 as a valid and a reliable tool to assess patient QoL. The EORTC QLQ-C30 concentrates on a patients' ability to fulfill the activities of daily life justifying its use in clinical trials investigating new drugs or novel combinations of agents. Clinical practitioners and investigators need to know what happens to a patient's capacity to fulfill the activities of daily life at work and in the home. Consequently, this instrument has an extensive physical functioning scale coupled with a comprehensive symptom inventory.

There are three key findings of our study. First, appetite loss and global health at baseline provides prognostic information for survival after adjusting for the effects of age, gender, treatment history, tumor stage and other QoL variables. Second, improvement in physical function at 3 months is an indicator of improved patient survival after adjusting for other covariates. Third, contrary to what one might predict, improvement in social function at 3 months is independently associated with a worse survival.

Our finding of improvement in physical function scores correlating with better survival in CRC is consistent with recent studies in esophagogastric and head and neck cancer patients (HNC) [11,13]. In patients with localized HNC, Meyer F et al. found that at 1 year after treatment, the HR associated with a positive physical function change of 10 points was 0.75 (95% CI, 0.68 to 0.83). After physical function was taken into account, no other QoL variable was associated with survival [11]. In patients with esophagogastric cancer, a 10-point change in physical function (hazard ratio [HR], 0.85; 95% CI, 0.76 to 0.96; *P *= .007), pain (HR, 1.20; 95% CI, 1.09 to 1.33; *P *< .001), and fatigue (HR, 1.16; 95% CI, 1.04 to 1.30; *P *= .009) scores were each associated with better survival [13].

An explanation for the unexpected association of an increase in the social function scale score and decreased patient survival cannot be elucidated from this study. Multicollinearity does not seem to explain this counterintuitive finding. It is relevant to note, however, that the two questions that comprise this scale query both the effects of physical condition and medical treatment on social function. Thus, both factors contribute to the overall social function scale score but are expected to be weighted differently at each assessment point for any individual patient. Since this function is reported as a single score, it is impossible to delineate the impact of each factor on the change score. Nevertheless, it is reasonable to speculate that change in the social function score that is caused primarily by the effects of medical treatment would be of lower prognostic value than changes in physical condition. This hypothesis is testable and worth further investigation. Our unexpected finding regarding the social function scale stands in contrast with the finding reported by Efficace et al. in advanced CRC, where a 9% decrease in patient's hazard of death was found for any 10-point increase in the social functioning score. In that study, social functioning was concluded to be a prognostic measure of survival beyond a number of previously known biomedical parameters [28]. This finding was further validated in an independent sample of metastatic CRC patients by the same research group [18].

The results of this study have important implications for both clinical and research practices. They suggest that baseline QoL should be considered when planning treatment and regular QoL assessment performed during the course of treatment. Furthermore, interventions aimed at improving specific QoL parameters should be applied when indicated. The utility of this approach to patient management, based on the findings described in this study, would be validated definitively if interventions that enhance specific QoL parameters are shown to enhance survival.

Thus, the findings reported here suggest that QoL monitoring, coupled with treatment to improve appetite loss, global health and physical function when indicated, should be investigated in prospective studies in CRC. Positive effects on survival as a consequence of interventions designed specifically to improve patient symptoms and QoL independent of tumor therapy would go a long way towards establishing causative relationships between specific QoL parameters and disease control. Although some progress has been made with respect to the treatment of appetite loss and physical function in cancer patients, clinical effectiveness is inconsistent and unpredictable. And there are at present no effective means to address more complex QoL factors such as global health. This challenges the cancer research enterprise to develop greater understanding of the complex physiology responsible for all aspects of QoL, and to use this information to develop more effective and predictable methods to favorably modulate this critical aspect of patient health and wellness.

Several limitations of this study require careful acknowledgment. Our study, because of its retrospective nature, relies on data not collected to test a specific hypothesis. As a result, we could not control for certain factors in our analyses that could influence survival such as treatment received at our institution, medical co-morbidities, socioeconomic factors, support system, exercise and educational level. The patient cohort was limited only to those patients who were English speakers and therefore is not representative of the complete spectrum of colorectal cancer patients. Moreover, this study does not reveal a causative relationship between QoL and survival. Rather, patient QoL was found to act as a surrogate for otherwise undetected prognostic factors [1]. QoL scores were assessed over a three month interval only which may not be sufficient time for score changes to develop in other QoL parameters that may be prognostic of survival. We did not control for the multiple comparisons made in this study, but this is acceptable for hypothesis-generating studies [10].

This study also has several strengths, including no missing data on any EORTC QLQ-C30 variables for the entire study sample; a homogeneous population of patients with advanced CRC (stages III and IV) at presentation to our hospitals; the use of a valid and reliable QoL instrument; the availability of clinical parameters in nearly all patients; and availability of mature and reliable survival data. As is the case for all exploratory retrospective studies, the most important outcome that can be achieved is the development of a hypothesis suggested by the results. As a consequence of this study, we hypothesize that the parameters of physical function, appetite loss, and global health are independent determinants of survival in colorectal cancer, and should be regularly assessed and when indicated, targeted for intervention.

## Conclusions

This exploratory study provides preliminary evidence to indicate that CRC patients whose physical function improves within 3 months of treatment have a significantly increased probability of survival. These findings should be used in clinical practice to systematically address QoL-related problems of CRC patients throughout their treatment course.

## Competing interests

The authors declare that they have no competing interests.

## Authors' contributions

DPB and DG participated in concept, design, data collection, data analysis, data interpretation and writing. JFG participated in data analysis, data interpretation and writing. EDS participated in concept, design, data interpretation and writing. All authors read and approved the final manuscript.

## References

[B1] Coates A Porzsolt F Osoba D Quality of life in oncology practice: prognostic value of EORTC QLQ-C30 scores in patients with advanced malignancy Eur J Cancer 1997 33 1025 1030 10.1016/S0959-8049(97)00049-X 9376182

[B2] Collette L van Andel G Bottomley A Oosterhof GO Albrecht W de Reijke TM Fossà SD Is baseline quality of life useful for predicting survival with hormone-refractory prostate cancer? A pooled analysis of three studies of the European Organisation for Research and Treatment of Cancer Genitourinary Group J Clin Oncol 2004 22 3877 3885 10.1200/JCO.2004.07.089 15459209

[B3] Dancey J Zee B Osoba D Whitehead M Lu F Kaizer L Latreille J Pater JL Quality of life scores: an independent prognostic variable in a general population of cancer patients receiving chemotherapy. The National Cancer Institute of Canada Clinical Trials Group Qual Life Res 1997 6 151 158 9161115 10.1023/a:1026442201191

[B4] Dharma-Wardene M Au HJ Hanson J Dupere D Hewitt J Feeny D Baseline FACT-G score is a predictor of survival for advanced lung cancer Qual Life Res 2004 13 1209 1216 15473499 10.1023/B:QURE.0000037481.36604.eb

[B5] Efficace F Biganzoli L Piccart M Coens C Van Steen K Cufer T Coleman RE Calvert HA Gamucci T Twelves C Fargeot P Bottomley A EORTC-BCG-IDBBC-NDDG Baseline health-related quality-of-life data as prognostic factors in a phase III multicentre study of women with metastatic breast cancer Eur J Cancer 2004 40 1021 1030 10.1016/j.ejca.2004.01.014 15093577

[B6] Langendijk H Aaronson NK de Jong JM ten Velde GP Muller MJ Wouters M The prognostic impact of quality of life assessed with the EORTC QLQ-C30 in inoperable non-small cell lung carcinoma treated with radiotherapy Radiother Oncol 2000 55 19 25 10788684 10.1016/s0167-8140(00)00158-4

[B7] Maisey NR Norman A Watson M Allen MJ Hill ME Cunningham D Baseline quality of life predicts survival in patients with advanced colorectal cancer Eur J Cancer 2002 38 1351 1357 10.1016/S0959-8049(02)00098-9 12091066

[B8] Montazeri A Milroy R Hole D McEwen J Gillis CR Quality of life in lung cancer patients: as an important prognostic factor Lung Cancer 2001 31 233 240 10.1016/S0169-5002(00)00179-3 11165402

[B9] Quinten C Coens C Mauer M Comte S Sprangers MA Cleeland C Osoba D Bjordal K Bottomley A EORTC Clinical Groups Baseline quality of life as a prognostic indicator of survival: a meta-analysis of individual patient data from EORTC clinical trials Lancet Oncol 2009 10 865 871 10.1016/S1470-2045(09)70200-1 19695956

[B10] Roychowdhury DF Hayden A Liepa AM Health-related quality-of-life parameters as independent prognostic factors in advanced or metastatic bladder cancer J Clin Oncol 2003 21 673 678 10.1200/JCO.2003.04.166 12586805

[B11] Meyer F Fortin A Gelinas M Nabid A Brochet F Tetu B Bairati I Health-related quality of life as a survival predictor for patients with localized head and neck cancer treated with radiation therapy J Clin Oncol 2009 27 2970 2976 10.1200/JCO.2008.20.0295 19451440

[B12] Luoma ML Hakamies-Blomqvist L Sjostrom J Pluzanska A Ottoson S Mouridsen H Bengtsson NO Bergh J Malmström P Valvere V Tennvall L Blomqvist C Prognostic value of quality of life scores for time to progression (TTP) and overall survival time (OS) in advanced breast cancer Eur J Cancer 2003 39 1370 1376 10.1016/S0959-8049(02)00775-X 12826039

[B13] Djarv T Metcalfe C Avery KN Lagergren P Blazeby JM Prognostic value of changes in health-related quality of life scores during curative treatment for esophagogastric cancer J Clin Oncol 2010 28 1666 1670 10.1200/JCO.2009.23.5143 20194863

[B14] Oskam IM Verdonck-de Leeuw IM Aaronson NK Kuik DJ de Bree R Doornaert P Langendijk JA Leemans RC Quality of life as predictor of survival: a prospective study on patients treated with combined surgery and radiotherapy for advanced oral and oropharyngeal cancer Radiother Oncol 2010 97 258 262 10.1016/j.radonc.2010.02.005 20189668

[B15] Blazeby JM Brookes ST Alderson D The prognostic value of quality of life scores during treatment for oesophageal cancer Gut 2001 49 227 230 10.1136/gut.49.2.227 11454799 PMC1728414

[B16] Camilleri-Brennan J Steele RJ Prospective analysis of quality of life and survival following mesorectal excision for rectal cancer Br J Surg 2001 88 1617 1622 10.1046/j.0007-1323.2001.01933.x 11736975

[B17] Earlam S Glover C Fordy C Burke D Allen-Mersh TG Relation between tumor size, quality of life, and survival in patients with colorectal liver metastases J Clin Oncol 1996 14 171 175 8558193 10.1200/JCO.1996.14.1.171

[B18] Efficace F Innominato PF Bjarnason G Coens C Humblet Y Tumolo S Genet D Tampellini M Bottomley A Garufi C Focan C Giacchetti S Lévi F Chronotherapy Group of the European Organisation for Research and Treatment of Cancer: Validation of patient's self-reported social functioning as an independent prognostic factor for survival in metastatic colorectal cancer patients: results of an international study by the Chronotherapy Group of the European Organisation for Research and Treatment of Cancer J Clin Oncol 2008 20;26 2020 2026 18421055 10.1200/JCO.2007.12.3117

[B19] Lis CG Gupta D Granick J Grutsch JF Can patient satisfaction with quality of life predict survival in advanced colorectal cancer? Support Care Cancer 2006 14 1104 1110 10.1007/s00520-006-0100-3 16819630

[B20] Osoba D Rodrigues G Myles J Zee B Pater J Interpreting the significance of changes in health-related quality-of-life scores J Clin Oncol 1998 16 139 144 9440735 10.1200/JCO.1998.16.1.139

[B21] Aaronson NK Ahmedzai S Bergman B Bullinger M Cull A Duez NJ Duez NJ Filiberti A Flechtner H Fleishman SB de Haes JC The European Organization for Research and Treatment of Cancer QLQ-C30: a quality-of-life instrument for use in international clinical trials in oncology J Natl Cancer Inst 1993 85 365 376 10.1093/jnci/85.5.365 8433390

[B22] Groenvold M Klee MC Sprangers MA Aaronson NK Validation of the EORTC QLQ-C30 quality of life questionnaire through combined qualitative and quantitative assessment of patient-observer agreement J Clin Epidemiol 1997 50 441 450 10.1016/S0895-4356(96)00428-3 9179103

[B23] Hjermstad MJ Fossa SD Bjordal K Kaasa S Test/retest study of the European Organization for Research and Treatment of Cancer Core Quality-of-Life Questionnaire J Clin Oncol 1995 13 1249 1254 7738629 10.1200/JCO.1995.13.5.1249

[B24] Van Steen K Curran D Kramer J Molenberghs G Van Vreckem A Bottomley A Multicollinearity in prognostic factor analyses using the EORTC QLQ-C30: identification and impact on model selection Stat Med 2002 21 3865 3884 10.1002/sim.1358 12483772

[B25] O'Brien Robert M A Caution Regarding Rules of Thumb for Variance Inflation Factors Quality & Quantity 2007 41 673 690 10.1007/s11135-006-9018-621815266

[B26] Besley D Kuh E Welsch R Regression Diagnostics: Identifying Influential Data and Sources of Multicollinearity 2004 Wiley, New York

[B27] Sauerbrei W Schumacher M A bootstrap resampling procedure for model building: application to the Cox regression model Stat Med 1992 11 2093 2109 10.1002/sim.4780111607 1293671

[B28] Efficace F Bottomley A Coens C Van Steen K Conroy T Schoffski P Schmoll H Van Cutsem E Köhne CH Does a patient's self-reported health-related quality of life predict survival beyond key biomedical data in advanced colorectal cancer? Eur J Cancer 2006 42 42 49 10.1016/j.ejca.2005.07.025 16298522

